# A Case of Gastric Cancer With Endoscopic Intact Gastric Mucosa

**DOI:** 10.7759/cureus.80424

**Published:** 2025-03-11

**Authors:** Akito Furuta, Shoji Oura, Naoki Kataoka

**Affiliations:** 1 Department of Gastroenterology, Kishiwada Tokushukai Hospital, Kishiwada, JPN; 2 Department of Surgery, Kishiwada Tokushukai Hospital, Kishiwada, JPN

**Keywords:** barrett’s esophageal cancer, esophageal adenocarcinoma, gastric cancer, heterotopic gastric gland, intact gastric mucosa

## Abstract

A 79-year-old man with histologically proven adenocarcinoma cells in the cardia was referred to our hospital for the treatment of gastric cancer. A repeat endoscopy, however, showed intact gastric mucosa at the cardia but pathologically showed adenocarcinoma cells in a slightly depressed esophageal lesion adjacent to the stomach. The patient, therefore, underwent endoscopic submucosal dissection (ESD) both to the distal esophageal and presumed cardia lesions to avoid under-treatment. Both ESD specimens, however, had no cancer cells. After careful endoscopic follow-up for 20 months, positron emission tomography showed a maximal standardized uptake value of 5.9 g/mL in the cardia, leading to the re-confirmation of adenocarcinoma cells in the cardia. The patient, therefore, underwent proximal gastrectomy and lymph node dissection. Postoperative pathological study showed cuboidal atypical cells forming irregular lumens and spreading widely in the submucosa with almost normal gastric and esophageal mucosa. Due to the lack of distinct submucosal heterotopic gastric glands in the stomach, this pathological condition seemed to have been caused by both the submucosal wide spreading of cancer cells and the loss of mucosal malignant foci. In conclusion, to avoid under-treatment, endoscopists should take this type of extremely rare gastric cancer with endoscopic intact gastric mucosa into consideration for differential diagnosis.

## Introduction

Endoscopes have markedly improved in resolution and function and have enabled physicians to detect more gastrointestinal cancers in their early stages. In addition, the advent of cure for gastrointestinal cancer only with minimally invasive endoscopic treatment, e.g., endoscopic submucosal dissection (ESD), has come to give great benefit to many patients with gastrointestinal malignancies [1].

Early gastric cancers themselves rarely exhibit clinical symptoms and are detected by endoscopy as protruding, superficial, or excavated lesions. Advanced gastric cancers present with various symptoms such as dysphagia, asthenia, and indigestion, and manifest endoscopically as polypoid, ulcerative, infiltrative ulcerative, or diffuse infiltrative tumors [2-4]. However, regardless of the stage or subtype, gastric cancers always have malignant cells in the gastric mucosa.

We herein report the case of an extremely rare gastric cancer having been under-treated due to both the presumed intact gastric mucosa on repeat endoscopy and no malignant cells in the ESD specimens despite the proven malignant cells in the stomach.

## Case presentation

**Figure 1:**
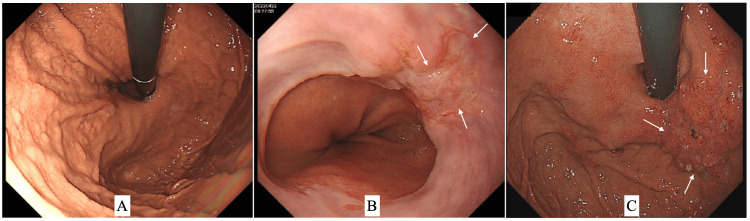
Endoscopic findings A repeat endoscopy showed an ulcerative lesion not in the stomach (A) but in the esophagus (B, arrows). Follow-up endoscopy finally showed a slightly elevated gastric lesion (C, arrows).

**Figure 2:**
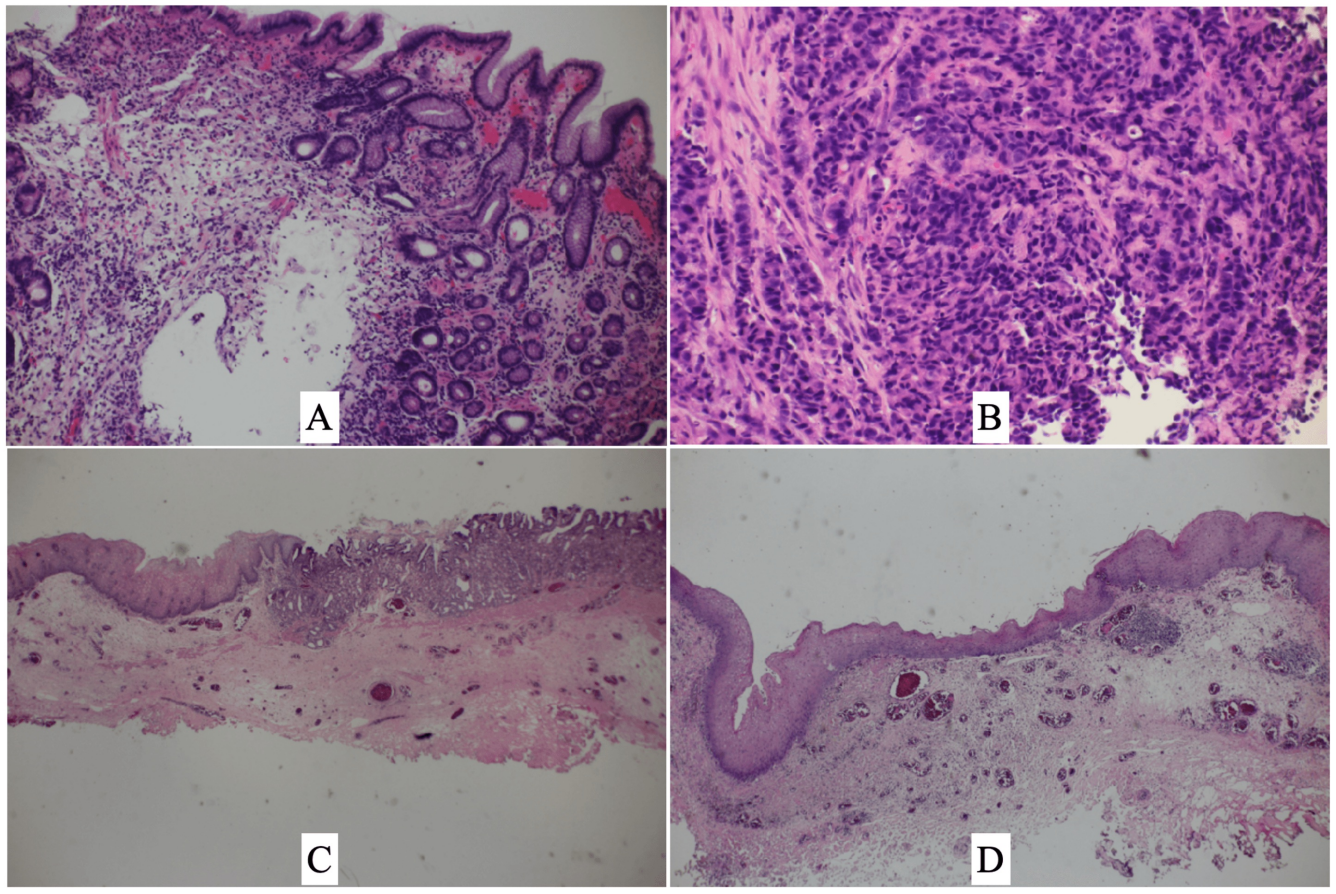
Pathological findings of the biopsy and ESD specimens Endoscopic biopsy specimens pathologically showed malignant findings not in the stomach (A) but in the esophagus (B). ESD specimens pathologically showed malignant cells neither in the cardia (C) nor in the esophagus (D). In addition, the esophageal ESD specimen had no Barrett esophagus (D). ESD, endoscopic mucosal dissection

A 79-year-old man with a history of *Helicobacter pylori *infection was referred to our hospital due to the histologically proven adenocarcinoma cells in the cardia on an annual checkup for his chronic gastritis at a private clinic. However, a repeat endoscopy at our hospital did not show any malignant findings in the gastric mucosa (Figures 1A, 2A) but pathologically showed adenocarcinoma cells in a slightly depressed lesion at the esophagus (Figures 1B, 2B) near the esophagogastric junction (EGJ).

Therefore, under the tentative diagnosis of Barrett’s esophageal cancer, the patient underwent ESD both to the esophageal and presumed gastric lesions to avoid under-treatment. Pathological study, however, showed no malignant cells both in the gastric and esophageal ESD tissues (Figures 2C, 2D) and made us carefully follow up the patient with periodical endoscopies.

**Figure 3:**
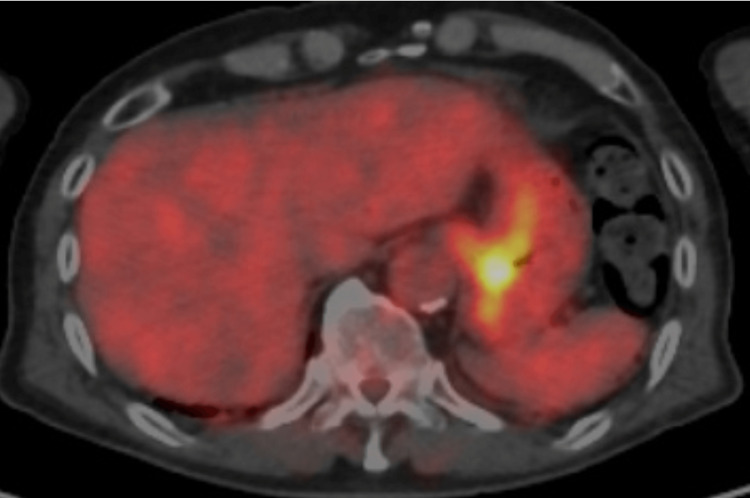
PET/CT findings PET/CT showed avid fluorodeoxyglucose uptake in the cardia 20 months after endoscopic submucosal dissection. PET/CT, positron emission tomography/computed tomography

After five negative endoscopic follow-ups during 20 months, positron emission tomography (PET) showed a maximal standardized uptake value of 5.9 g/mL in the cardia (Figure 3).

**Figure 4:**
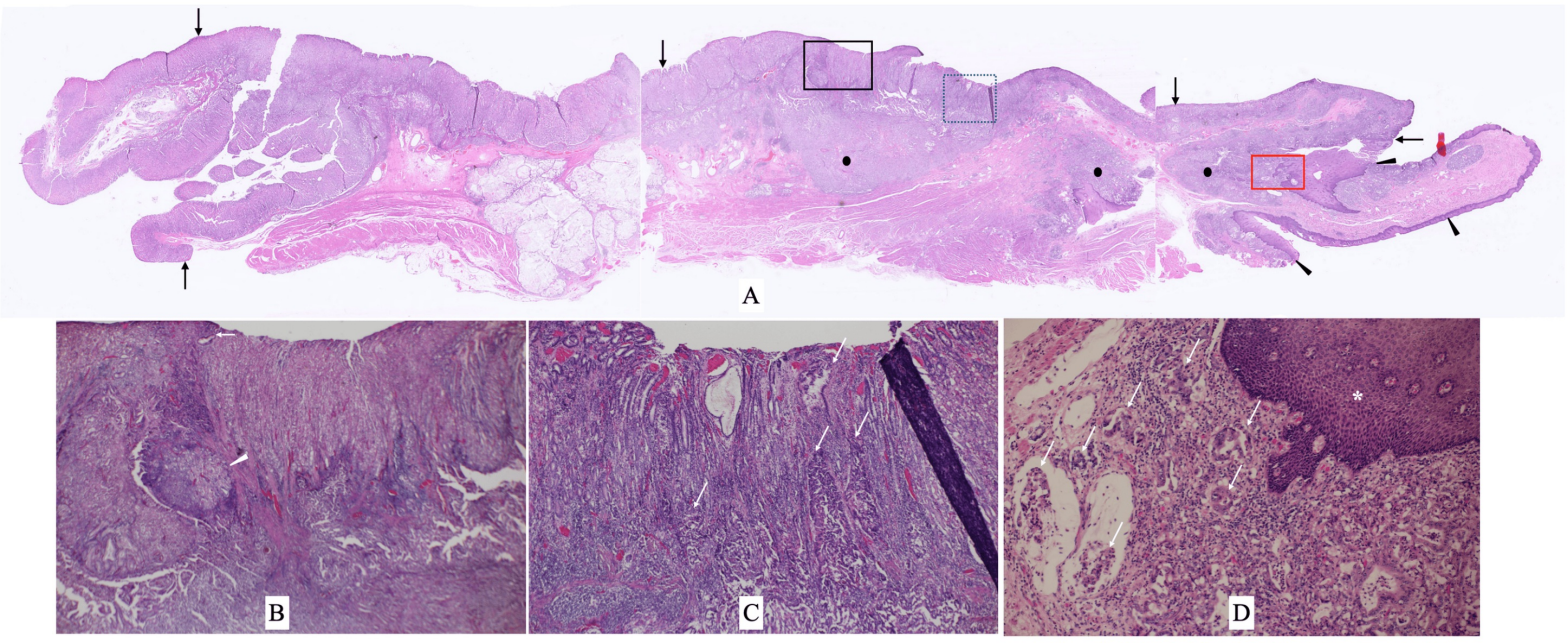
Pathological findings of the gastrectomy specimen (A) Gastric (arrows) and esophageal (arrowheads) mucosa had no exposed malignant cells. Cancer cell nests (closed circles) spread widely under the gastric and esophageal mucosa. (B) Magnified view of the black square in Figure A showed an oval large cancer cell nest (arrowhead) and very few cancer cell infiltrations close to the gastric mucosa (arrow). (C) Magnified view of the dotted square in Figure A showed a lot of cancer cell infiltration (arrows) very close to the gastric mucosa. (D) Magnified view of the red square in Figure A showed sparse distribution of adenocarcinoma cells (arrows) under the normal esophageal mucosa (asterisk).

In addition, endoscopic biopsy after the PET evaluation showed malignant adenocarcinoma cells in the slightly elevated cardia lesion very close to the EGJ (Figure 1C). The patient, therefore, underwent a proximal gastrectomy and lymph node dissection with additive resections both for oral and anal directions due to the positive margins by massive lymphatic vessel involvement on frozen section. Postoperative pathological study showed cuboidal atypical cells mainly forming irregular lumens and spreading widely in the submucosa with almost normal overlying gastric and esophageal mucosa (Figures 4A-4D), marked lymphatic vessel involvement, and three positive nodes.

Due to the lack of distinct submucosal heterotopic gastric glands in the stomach, this pathological condition seemed to have been caused by the disappearance of mucosal malignant foci. The patient unfortunately developed anastomotic failure and received various treatments such as a re-operation and endoscopic stenting to the anastomotic failure site but died of respiratory deterioration in approximately four months after the gastrectomy.

## Discussion

Endoscopic evaluation at our hospital showed no malignant findings in the gastric mucosa despite the presence of histologically proven adenocarcinoma cells in the same region at the private clinic. We, however, treated both the esophageal and presumed cardia lesions with ESD, the least invasive therapeutic option [5], to avoid under-treatment, unfortunately leading to confirming no malignant cells in the two ESD tissues. Some authors have reported the successful removal of submucosal heterotypic gastric glands or cancers originating in them [6-8]. The reason for no cancer cells in the two ESD tissues remains uncertain in this case.

A major clinical problem arose from the careless endoscopic follow-up only on the pathological report that stated that the ESD specimens had no cancer cells in this case. Retrospective pathological evaluation clarified the absence of Barrett's esophagus in the esophageal ESD specimen. In addition, retrospective endoscopic image evaluation did not show any Barrett's esophagus findings [9]. These facts suggest that the adenocarcinoma cells observed in the esophagus did not originate from Barrett's esophagus. Recognition of these facts, if had been shared with diagnostic team members, would have led us to another diagnostic approach.

Postoperative pathological study showed that the malignant cells proliferated widely and laterally under the gastric and esophageal submucosa and spread vertically and sparsely towards the gastric mucosa. In addition, despite the massive presence of malignant cells in the esophageal submucosa, no malignant cells were observed in the esophageal mucosa. These pathological findings, when combined with the absence of endoscopic malignant findings in the stomach on the initial repeat endoscopy at our hospital, strongly suggested that either malignant cells arose from ectopic gastric glands in the stomach or that malignant cells in the mucosa had disappeared after they spread widely in the gastric submucosa.

Heterotopic gastric glands in the stomach generally have cystically dilated gastric gland ducts in the gastric submucosa [7]. We have previously reported a case of gastric cancer arising from ectopic gastric glands in the stomach [10]. Our prior case also had very extensive submucosal spread of cancer cells and dilated normal gastric glands. We, however, could not find any cystically dilated gastric gland ducts in the gastric submucosa in this case. Meanwhile, we did not find any cancer cells exposed in the gastric mucosa. The mechanisms by which mucosal malignant lesions quasi-completely disappeared in this case are unclear. The most likely hypothesis is as follows. First, highly invasive cancer cells, at the time of forming only a very small lesion in the gastric mucosa, develop extensive submucosal spread. Second, mucosal lesions disappear through unknown mechanisms. Lastly, the ulcerated surface is covered by normal mucosa. This mechanism explains the pathological findings in this case. It is, however, naturally unclear why cancer cells do not remain in the mucosa at the ulcer edges. In addition, we cannot completely exclude the possible gastric cancer originating from ectopic gastric glands in the stomach based only on the absence of cystically dilated gastric gland ducts in the gastric submucosa.

## Conclusions

It is difficult to conclude whether gastric cancer in our case arose from ectopic gastric glands in the stomach or was a pathological condition as a result of disappearance of gastric mucosal malignant lesions. Endoscopic specialists, however, should note that some gastric cancers can develop this type of pathological condition. On suspecting these pathological conditions, physicians should apply endoscopic ultrasound to the submucosal lesions through presumed intact gastric mucosa to avoid under-treatment.
